# A comparative study of influenza surveillance systems and administrative data in England during the 2022–2023 season

**DOI:** 10.1371/journal.pgph.0003627

**Published:** 2024-09-20

**Authors:** Jonathon Mellor, Rachel Christie, James Guilder, Robert S. Paton, Suzanne Elgohari, Conall Watson, Sarah R. Deeny, Thomas Ward

**Affiliations:** 1 Data Analytics and Surveillance, UK Health Security Agency, London, United Kingdom; 2 Clinical and Public Health, UK Health Security Agency, London, United Kingdom; The University of Edinburgh, UNITED KINGDOM OF GREAT BRITAIN AND NORTHERN IRELAND

## Abstract

Accurate and representative surveillance is essential for understanding the impact of influenza on healthcare systems. During the 2022–2023 influenza season, the Northern Hemisphere experienced its most significant epidemic wave since the onset of the COVID-19 pandemic in 2020. Concurrently, new surveillance systems, developed in response to the pandemic, became available within health services. In this study, we analysed per capita admission rates from National Health Service hospital Trusts across four surveillance systems in England during the winter of 2022–2023. We examined differences in reporting timeliness, data completeness, and regional coverage, modelling key epidemic metrics including the maximum admission rates, cumulative seasonal admissions, and growth rates by fitting generalised additive models at national and regional levels. From modelling the admission rates per capita, we find that different surveillance systems yield varying estimates of key epidemiological metrics, both spatially and temporally. While national data from these systems generally align on the maximum admission rate and growth trends, discrepancies emerge at the subnational level, particularly in the cumulative admission rate estimates, with notable issues observed in London and the East of England. The rapid growth and decay phases of the epidemic contributed to higher uncertainty in these estimates, especially in regions with variable data quality. The study highlights that the choice of surveillance system can significantly influence the interpretation of influenza trends, especially at the subnational level, where regional disparities may mask true epidemic dynamics. Comparing multiple data sources enhances our understanding of the impact of seasonal influenza epidemics and highlights the limitations of relying on a single system.

## Introduction

The surveillance of influenza is crucial to monitor epidemiological trends and pressures on primary and secondary healthcare services [1, 2]. A key aspect of influenza surveillance is the monitoring of hospitalisation rates, which requires timely collection of the number of patients admitted to hospital and the population those admitted draw from. Monitoring admission rates is important as a measure of seasonal incidence, which enables timely operational response in healthcare systems.

Influenza is a transmissible respiratory virus that causes seasonal epidemics worldwide, resulting in significant morbidity, [3, 4] mortality [5], and increased pressure on healthcare systems [6, 7]. The influenza virus has 4 types, two of which, influenza A and B, tend to cause seasonal epidemics [8]. Common symptoms of influenza include fever, body aches and sore throats [9]. The disease can cause life threatening complications, with greatest risk for those over 65, under 2 years old, or with chronic health conditions [1, 10, 11]. Since the onset of the COVID-19 pandemic, influenza transmission was limited by the impacts of non-pharmaceutical-interventions [12–14], changes to contact networks [15], and increases in the size of the susceptible population [16], making a resurgent influenza epidemic wave of key importance for healthcare operational demands. The joint healthcare pressure of an influenza wave and a COVID-19 wave at the same time, co-circulating [17], posed an unseen scenario for care providers in 2022, with a considerable risk of overstretched intensive care unit capacities [18].

Influenza trends, and specifically hospitalisations with influenza, are measured and monitored in several ways across administrative data sources and active surveillance systems in England [2]. These data are collected for multiple purposes and available as a mix of public reports and within-health system internal monitoring. SARI-Watch is the primary system, reported publicly by the UK Health Security Agency (UKHSA), monitoring influenza admission rates [2], via a sentinel network in real time. However, there are three other data sources from which admission rates can be estimated. Some are real-time, such as counts of lab reported tests within the Second Generation Surveillance Service (SGSS) [19], or rapid hospital census’s of new influenza patients in the Urgent and Emergency Care (UEC) reports [20]. An alternative data source is the retrospective patient episode data used for managing hospital payments, the Secondary Use Services (SUS) database [21].

In this research we compare the four data sources of influenza hospitalisations over the winter 2022–2023 season in England and explore the different characterisations of the epidemic waves produced by modelling each data source. We produce estimates of key metrics including the size, timing, and growth rate of the influenza admissions per capita at national and National Health Service (NHS) regional geographies. Comparisons of surveillance systems are typically done across different settings (such as incidence in primary care, secondary care, and mortality), in this work we compare across data sources within a single setting.

## Methodology

This study compares different administrative and surveillance data sources for influenza admission rates, highlighting their key distinctions in measurement and purpose. The four data sources represent a comprehensive selection of methods used by healthcare and public health organisations in England to measure influenza admissions. We evaluate these data sources based on timeliness, coverage, completeness, and accuracy, employing a range of epidemiologically relevant metrics. To achieve this, we analyse the characteristics of the data and key epidemic metrics, adjusting for temporal precision and sample sizes.

### Surveillance data

#### Secondary uses service–All Patient Care (APC)

The Secondary Uses Services (SUS) data set is created from information collected for the activity cost undertaken whilst a patient attends hospital. The administrative data is published monthly and allows for additional data to be added to pre-existing patient records [22]. There are five data sets within the SUS database: Accident and Emergency, Admitted Patient Care (APC), Adult Critical Care, Outpatients and Maternity. The focus for this study is the SUS APC data, which is made available with a 2–3 month reporting lag. The data is measured by consultant episode and per NHS Trust, including geographical information about the treatment site, and patient information, such as age and patient residence. The data includes admission/discharge date, diagnosis, and procedure codes, covering all Integrated Care Boards in England [23, 24]. Influenza patients are identified using ICD-10 codes J09, J10 and J11 [25] as either primary or secondary diagnosis. Patients with COVID-19 emergency code (U071), or individuals who tested positive for COVID-19 between fourteen days before, and one day after admission were classified as COVID-19 patients and excluded to remove possible co-infections (a small fraction of admissions). The COVID-19 tests were linked from the UKHSA Second Generation Surveillance Service (SGSS) [19]. The APC data is not immediately complete as records may be backfilled following discharge, and diagnosis codes may not be recorded, with quality varying across NHS Trusts [21]. Data was obtained on the 28^th^ of August 2023. Patients who receive an influenza diagnosis should have tested positive for the disease based on the prescribed guidance [26], although this challenging to verify without direct data linkage between patient and test. This data source provides detailed information about the patient; however, it tells us little about the pathogen itself, with no typing or sub-typing information recorded.

#### SARI-Watch (SARI)

UKHSA SARI-Watch is a surveillance system designed to monitor respiratory disease hospitalisations. It includes weekly aggregate data for test confirmed patients with influenza, RSV or COVID-19 by discrete age ranges [27], as well as differentiation between general admission and critical care admission. The data contains subtyping information, such as which influenza A subtype has been detected, informing severity assessment and vaccine policy decisions. Moreover, SARI-Watch contains patients with clinical symptoms testing positive via point of care or laboratory tests [27]. The SARI-Watch Sentinel admissions data is collected from a subset of Trusts in England forming a sentinel network. In comparison to the passive approach of other data sources through administrative collection, the SARI data relies on recruiting participating Trusts and engagement to maintain collection quality. The SARI-Watch Sentinel influenza admissions data we explore in this study contains an ISO week identifier, Trust identifier, and admission counts for influenza stratified by age range and influenza sub-type or type. Sentinel Trusts are re-recruited annually, with some joining and leaving each year. Sites were initially recruited using stratified sampling to ensure a representative sample of the country [27]. This data is the accepted estimate of admissions rates, the UK Official Statistics, with a history of utility for monitoring respiratory illness in near-real-time for public health response.

#### National health service England Urgent and Emergency Care Situational Report (UEC)

The National Health Service England (NHSE) Urgency and Emergency Care (UEC) data is provided by individual NHS Trusts, delivering a daily situation report covering urgent and emergency care over the previous 24 hours [20]. The administrative reporting process for many Trusts is automated and submitted via web form [28]. Information on both hospital bed occupancy and patients in the past 24 hours who tested positive for influenza are shared weekly. For comparison, we assume newly test positive patients in the last 24 hours are analogous to daily admissions. This system was created as a wider situation report for more than influenza; however, we restrict this analysis to influenza. The data collection for influenza begun in 2021 and therefore does not contain historic influenza pre-COVID-19 comparison benchmarks. The data source provides aggregate test positive patient counts, without information of the disease itself, such as typing, sub-typing, or relevant patient characteristics like age.

### Second Generation Surveillance System (SGSS)

The Second Generation Surveillance System (SGSS) is an administrative database storing infectious disease test results in the UK [19]. Within the UK, influenza is a notifiable causative agent according to the Health Protection (Notification) regulations (2010) [29]. Both positive and negative tests were required to be reported within seven days by laboratories in 2022 [30]. The SGSS data was obtained on 12 September 2023. Within SGSS there are identifiers for the categories of test (such as a rapid test or PCR, the setting of the test (such as primary care, inpatient, or emergency care) and which organisation requested the test. We selected only tests requested by NHS Trusts, within an inpatient setting, and deduplicate the tests to be one per patient infection episode. This gives the date a patient first tested positive for influenza as an inpatient, and therefore a proxy for an influenza admission. This approach relies on complete records, where the NHS Trust and setting can be identified, which is not always the case. The completeness of fields used to link tests and the accuracy of these identifiers varies by NHS Trust and over time. The SGSS data provides some information on the disease itself, such as influenza A or B (where the test allows), and on patient characteristics such as age.

### Population catchment estimates

To determine admission rates per capita, giving estimates of admissions across disparate hospital groupings, we need a population denominator. NHS Trusts do not service a clearly defined population, with multiple providers placed within the same administrative regions. The hospital a patient attends emergency care depends on location and choice [31], with health seeking behaviour varying across age groups [32].

To calculate the effective population of an NHS Trust we produced a proportionate mapping between NHS Trust and lower-tier local authorities (LTLA) utilising the same approach from the Office of Health Improvement and Disparities NHS Acute (Hospital) Trust Catchment Population experimental statistics [33]. We queried the APC database for all admitted patients then aggregated by Trust and residential LTLA to produce the proportionate mapping.

With this mapping, we used the ONS 2019 local authority population estimates to produce a weighted sum of populations across Trusts of their feeder LTLAs, giving an effective population catchment size for each hospital Trust.

### Processing

Each dataset is transformed into a count of influenza admissions per day and by NHS Trust that reported to the respective system, to allow for comparison. The population estimate for each NHS Trust is joined to the transformed data. We define the influenza season as the 2^nd^ of October 2022 to the 21^st^ of May 2023, epidemiological weeks 40 to 20.

The SARI-Watch data are available as weekly counts and therefore, we divide by 7 to convert to daily granularity. As the collection is Monday-Sunday, the date is selected as the midpoint, Thursday. The NHS Trusts included are acute secondary care providers defined in the Estates Returns Information Collection data [34], with specialist Trusts removed. As the different sources are a mix of opt-in surveillance (SARI), administrative secondary purpose collections (SUS, SGSS) and a newly set up reporting system (UEC), there is variation in Trust participation. We define inclusion criteria for Trusts to ensure a minimum level of quality, reducing bias in the estimates. Trusts are excluded from the analysis using the following criteria:

1. The Trusts reported missing values over the whole study period (aggregate reports count: UEC, SARI).
2. The Trust reported a count of 0 for more than 90% of dates in the study period (aggregate reports: UEC SARI). The Trust did not report a test or admission for over 90% of dates in the study period (individual reports: APC, SGSS).

Criteria 1 is used to remove non-participating Trusts in aggregate reporting and criteria 2 is used to remove incorrect null returns, which bias the admission rate downward and Trusts not providing identifier details in individual records, such as an organisation code. The choice in threshold value for criteria 2 is shown in Fig A in S1 Text.

### Ethics statement

The data collected for this study were explicitly collected for the purpose of surveillance, or routinely collected administrative data for healthcare management. Personal data were anonymised in secure systems to prevent linkage or identification, and only accessed by those with appropriate accreditation and permission. The analysis did not require the use of sensitive patient characteristics, therefore, these were removed from data at the earliest possible point. Data were aggregated to coarse spatial scales, with large populations, further reducing the risk of identification and sensitive characteristics.

UKHSA have an exemption under regulation 3 of section 251 of the National Health Service Act (2006) to allow identifiable patient information to be processed to diagnose, control, prevent, or recognise trends in, communicable diseases and other risks to public health.

### Model

To infer characteristics of the epidemic wave, we need to quantify uncertainty due to sample sizes and adjust for reporting effects. Three of the four data sources within the analyses are daily, and therefore day-of-week effects tests consistently lower over weekends compared to the weekdays. We estimated the admissions per capita as a smooth function of time (*s*(*t*)) using a generalised additive model (GAM). The GAM used thin-plate splines through time with a negative binomial error structure, log-link function and model offset for the population size. A random effect *f*_2_ was used to adjust for the day-of-week effects (dow) in reporting for APC, UEC and SGSS data sources, which was not required for the weekly SARI data. The thin-plate spline *f*_1_ is fit over time points *t*. The national models are defined in Eqs 1 and 2, with the regional models defined in Eqs 3 and 4, where *i* denotes the region, producing an independent spline fit for each region, with a cross region pooled day-of-week adjustment. The population size is given as population size(*t*, *i*) which is the sum of Trust catchment populations that reported admissions at *t*.


$$s(t)≔\log\left(admissions(t)\right)=\beta_{0}+f_{1}(t)+f_{2}(dow)+log(population\;size(t))$$ Eq 1

$$s(t)≔\log\left(admissions(t)\right)=\beta_{0}+f_{1}(t)+log(population\;size(t))$$ Eq 2

$$s(t)≔log(admissions(t))=\beta_{0}+f_{1}(t,i)+f_{2}(dow)+log(population\;size(t,i))$$ Eq 3

$$s(t)≔\log\left(admissions(t)\right)=\beta_{0}+f_{1}(t,i)+log(population\;size(t,i))$$ Eq 4

The models are fit using the R package *mgcv* [35]. We extracted samples of the model fit to quantify both model and data uncertainty, and the growth rate, using a central finite difference approach with the R package *gratia* [36]. By taking the estimated smooth function of time *s*(*t*), its derivative the instantaneous growth rate $\frac{d\;s(t)}{dt}$, we calculate the instantaneous doubling time as $\frac{log(2)}{\frac{d\;s(t)}{dt}}$. From the fit models, we generate 1000 posterior samples for the admissions per capita and growth rate via an approximate multivariate normal method, using *gratia*.

### Comparison metrics

Day-of-week effects in admissions confound the true underlying epidemic growth rate. Since these are modelled as random effects in Eqs 1 and 2, we omit these weekly cycles in admission numbers from our estimated per capita admission rates and growth rates. From the admission rate and growth rate posterior samples we calculate summary statistics across the sample draws and use quantiles to capture the estimate’s uncertainty. For each summary metric of the epidemic wave by data source, we produced a median estimate and 95% confidence interval.

To characterize the epidemic wave, we produce estimates of key metrics of interest for the size, timing, and growth rate of influenza admissions. The maximum admission rate is the maximum value of each set of posterior draws across the wave. The peak date is the date at which the maximum admission rate occurs across the posterior draws. The cumulative admission rate is the sum of the daily admission rate over the wave for each set of posterior draws. We also perform inference on qualities of the epidemic wave growth rate, extracting the maximum value, timings of change points, and the length of the peak, defined as the time between the maximum and minimum growth rates.

## Results

### Timeliness

The availability of data in real-time is an important consideration when analysing trends within-season. The reporting frequency and lag are given in Table 1. The APC reporting lag is up to 3 months (with variation between Trusts), making it impractical to use in real-time. Both the UEC and SARI data are shared once per week, up to the most recent completed week, which is often sufficient for decision making. Some Trusts may revise their reports, adjusting rates retrospectively for recent weeks. As a testing database SGSS is available daily, though there is often backfilling in the most recent days. The daily reports of APC, SGSS and UEC give more temporal information than the weekly SARI, though this higher granularity is subject to higher stochasticity and day-of-week effects.

**Table 1:** Definition of an influenza admission across APC, UEC, SARI and SGSS data. Admissions are considered as patients admitted into the acute hospital in either a general ward or critical care. Both PCR and molecular point of care tests are included. We define the reporting frequency as how often information is shared, and the reporting lag as the time between information submission and the information being complete.

Data Source (Label)	Influenza admission definition	Coding system	Collection type	Granularity	Reporting Frequency	Reporting Lag
Secondary Use Services–Admitted Patient Care (APC)	Patient admitted to hospital with a primary or secondary diagnosis code of influenza. Excluding patients that have a primary or secondary diagnosis of COVID-19 or positive COVID-19 test within 14 days of admission.	ICD-10 code	Administrative	Individual (patient characteristics and hospital episode information)	Daily	Approximately 3 months
NHS England Urgent and Emergency Care Situational Report (UEC)	New inpatients in the past 24 hours with a laboratory confirmed positive influenza test. This will include both admissions due to influenza, and patients with influenza.	Positive test	Administrative	Aggregate	Daily	< 1 week
SARI-Watch Sentinel (SARI)	Patient admitted to hospital which meets clinical symptom criterion and has a confirmed positive influenza test.	Positive test + symptoms	Survey	Aggregate (stratified by type, sub-type and age)	Weekly	< 1 week
Second Generation Surveillance System (SGSS)	First positive test for an individual while an inpatient within a hospital.	Positive test	Administrative	Individual (patient and test type information)	Daily	< 1 week

### Coverage

**Fig 1:**
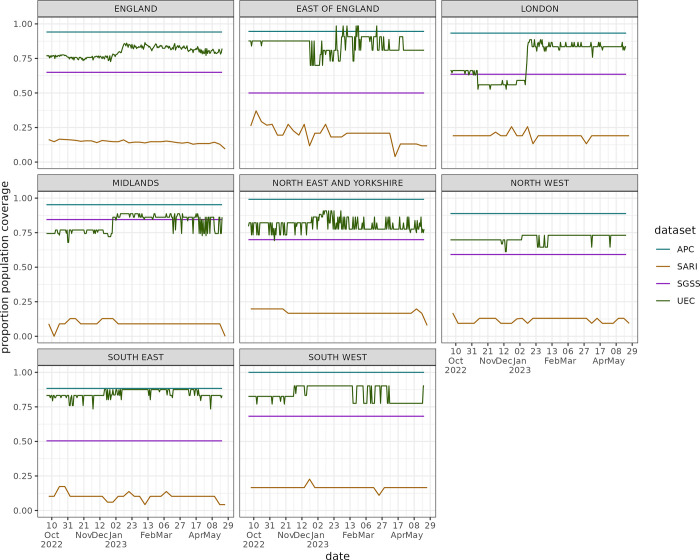
The population catchment of trusts reporting to each data source over time as a proportion of the total population in that geography. As individual level data sources, APC and SGSS do not change over time and are lower than 1.0 due to the inclusion criteria for reporting.

As hospital Trust participation evolves over time in some data sets, the population denominator is subject to change. The national count of Trusts reporting, and average population coverage over the season are presented in Table 2, along with raw counts of the peak and cumulative admissions, to demonstrate the scale of data source, unadjusted for reporting variation. Regional breakdowns are given in Table A in S1 Text. How the proportion of a population covered by the dataset changes through time is shown in Fig 1. APC consistently reports the highest coverage, at near 100% population catchment reporting, though not in real-time, with a three-month reporting lag. In England, the next highest, UEC, covers approximately 80% of the population. This data collection is the newest, with some Trusts coming online mid-season, increasing the coverage. This proportion changes with time, with more Trusts starting to report part way through the season after the peak had passed, driven by the Midlands and London regions. Regionally, SGSS has reporting from 50% population coverage and above, although there is high heterogeneity. This proportion is driven by missing information in the database, causing exclusions due to criteria 2 –Trusts not reporting tests over 90% of the season. The SARI dataset has lowest coverage, which is expected from a sentinel surveillance approach aiming for a representative sample of Trusts.

**Table 2:** Unmodelled national summary of reported data across the different data sources. Counts are from the processed data after exclusion criteria are applied. Trust reporting counts are the lowest and highest number of participating Trusts for a given report post exclusion criteria. The mean population coverage is taken as the mean national proportion of the population over the time series. Peak admissions are taken as the maximum admissions in each report and cumulative admissions the sum of all admissions reported. These metrics are not corrected for time varying participation and population catchment sizes. Regional breakdowns are given in Table A in S1 Text. The reporting frequency and lag are given based on approximate completeness delays.

Dataset	Trusts reporting (minimum–maximum)	Mean Population Coverage (%)	Peak Admissions	Cumulative Admissions
**APC**	115–115	94.1	1505	46043
**SARI**	12–19	14.5	227	1236
**SGSS**	78–78	65.0	1088	29606
**UEC**	89–105	79.7	1028	32706

### Completeness

The population covered by a system is informative, but so is the incidence it captures, which we refer to as completeness. All systems capture a fraction of the cumulative admissions recorded in APC, shown in Table 2. Naturally, sentinel surveillance (SARI) is not expected to be complete, but by comparing against APC we can understand the extent of the completeness. The counts over time across data are given in Fig B in S1 Text. The magnitudes of counts are broadly as expected when considering the population coverage in Fig 1, such as SARI completeness being low, with regional variation. However, in London the SARI counts are higher than those reported to UEC and SGSS, despite higher coverage from these sources.

### Accuracy

#### Modelled admissions and doubling time

**Fig 2:**
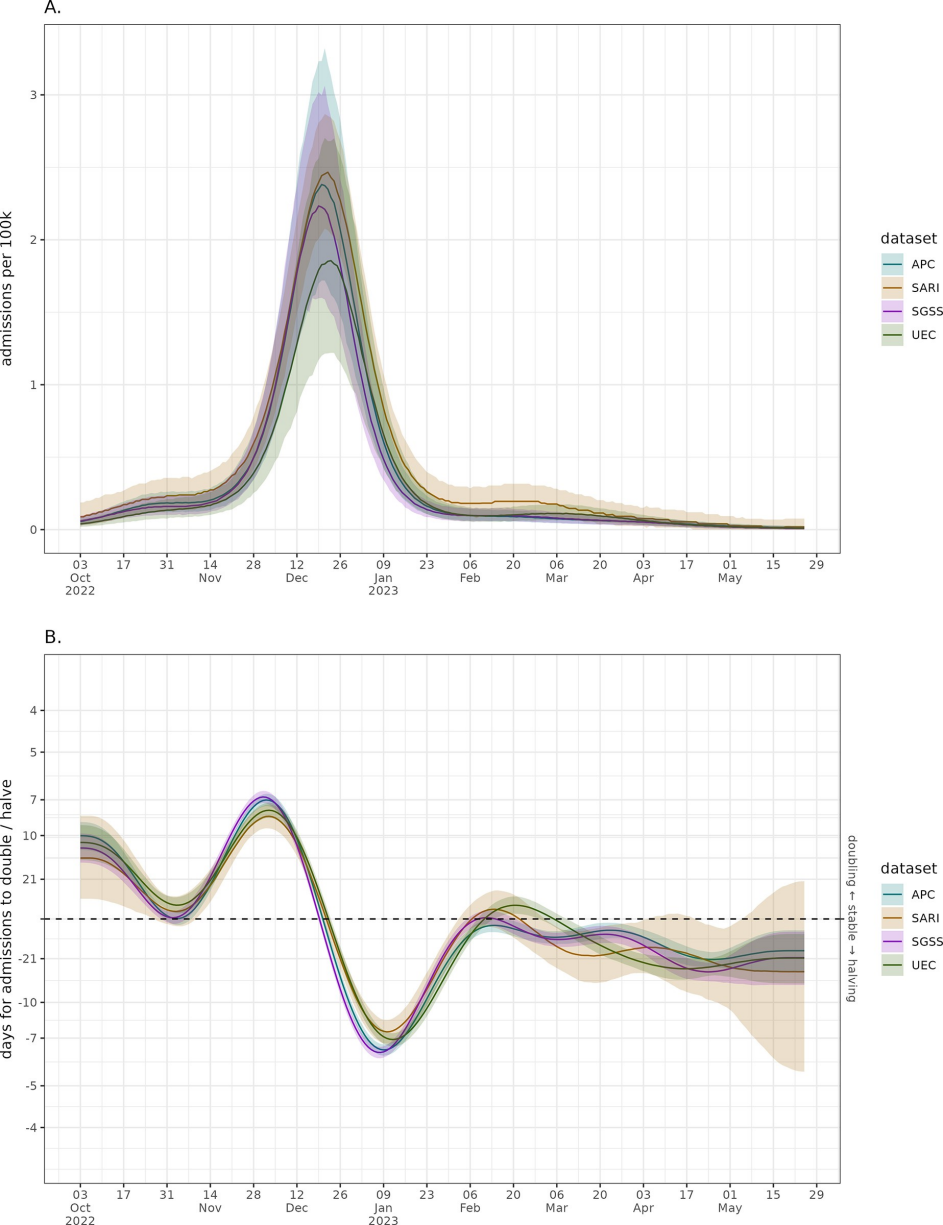
National modelled admission per capita wave with weekly effect correction (sub-plot A) and the national admissions growth rate expressed as a doubling/halving time (sub-plot B) across the different data sources. Solid lines represent median model estimate and ribbons the 95% confidence interval.

The winter 2022–2023 seasonal influenza wave was early in the season, with fast growth followed by sharp decay. The modelled national admissions wave timing appears to correspond well between datasets shown in Fig 2A, though the magnitudes of the rates differ. There is high uncertainty in the admission rate for UEC, with SARI having consistently higher values outside of the peak wave period. There is a similar picture of agreement in the doubling times for the different datasets in Fig 2B. Each source is growing at a similar rate and had a peak doubling time of approximately seven days. The unmodelled data with clear day-of-week effects and stochastic noise can be found in Fig C in S1 Text. There is higher uncertainty in the data sources with smaller samples, which has a large impact on the growth rate estimates.

**Fig 3:**
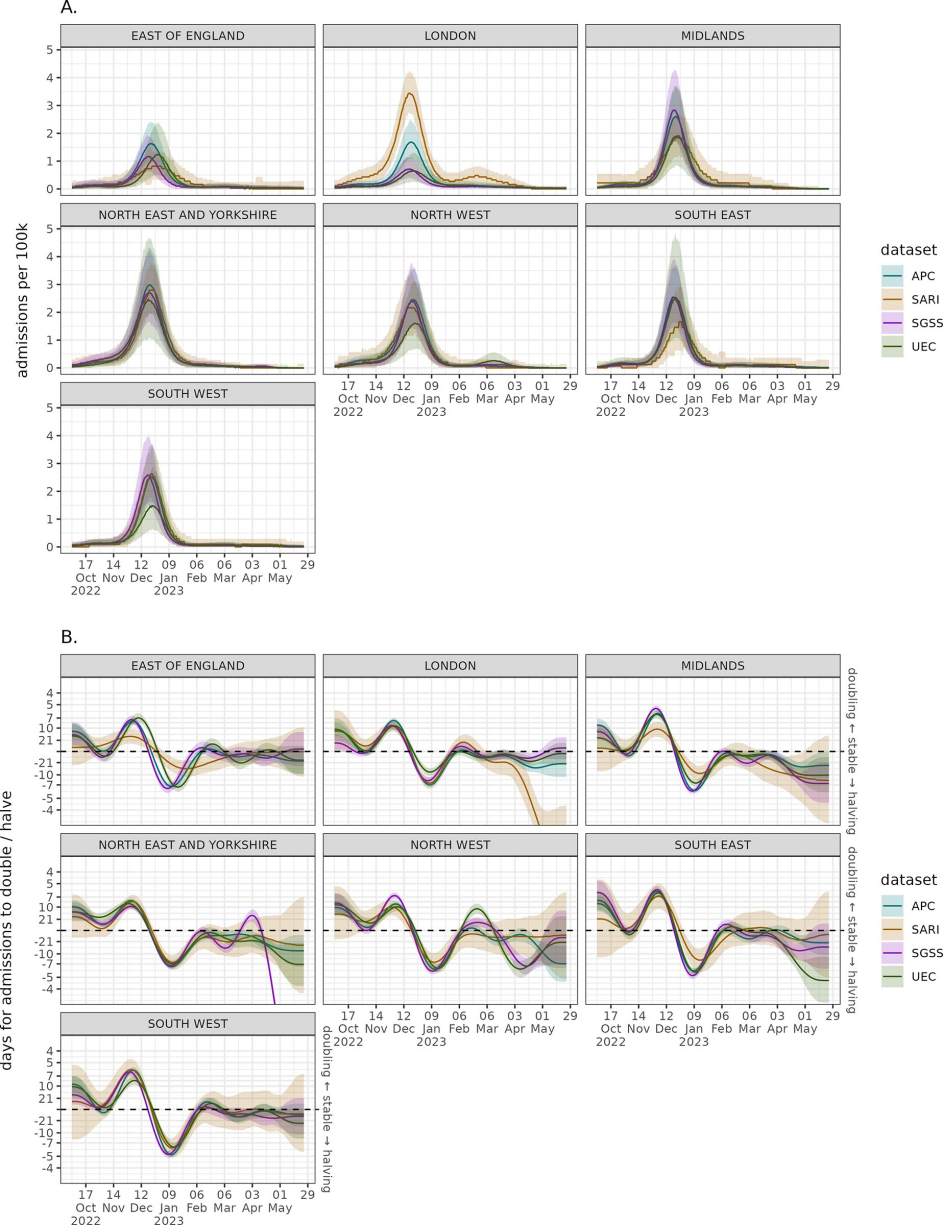
Regional modelled admission per capita wave with weekly effect correction (sub-plot A) and the regional admissions growth rate expressed as a doubling/halving time (sub-plot B) across the different data sources. Solid lines represent median model estimate and ribbons the 95% confidence interval.

The estimated regional per capita rates in Fig 3A broadly show agreement in trend across the data sources. The exception is London, where SARI estimates a notably higher rate with a gap between confidence intervals for much of the peak. UEC and SGSS estimated admission rates are substantially below the other two sources and appear to be lower than the other regions. The APC estimate in London is between these two different trends, and more similar to other regions. While it is important not to over interpret growth rates at low counts (the early and late season) there is regional uncertainty across datasets in Fig 3B in the beginning and at the end of the season. This high uncertainty in the growth rate means it takes longer to determine when a growth phase has started, when the confidence interval no longer overlaps a doubling time of zero. As expected from the smaller sample size approach there is more uncertainty in the SARI growth rate, though the direction agrees with other data.

#### Epidemic wave peak

**Fig 4:**
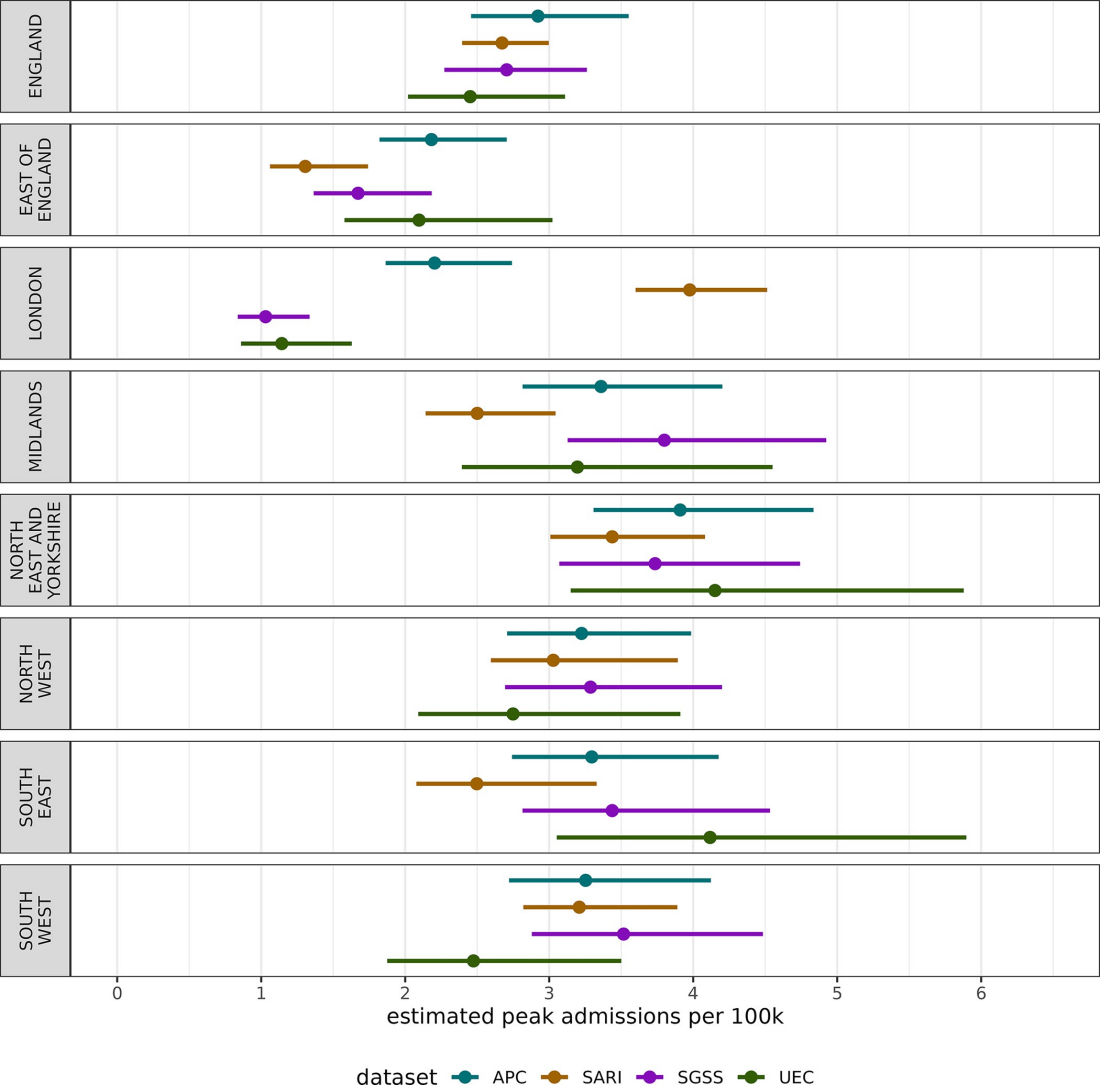
The estimated maximum number of admissions per capita nationally and regionally across each data source at the peak of the epidemic wave for the winter 2022–2023 season. The central point represents the median estimate, and the lines the 95% confidence interval.

The peak admissions rate of an influenza wave is a key characteristic when comparing seasons, but as shown in Fig 4 there is variation in peak admissions rate estimates regionally. Nationally, there is agreement on the maximum rate, with central estimates all between 2.4 and 3 admissions per 100k with overlapping confidence intervals. There is high uncertainty in the estimate produced by UEC across most regions, and as with other analyses, we can see a disparity in datasets for estimating the peak in London. Excluding London and the East of England there is agreement in the magnitude of the peak implying there may be different ascertainment rates or reporting in London and East of England. The timing of epidemic peak is given in Fig D in S1 Text, showing similar timings around 22 December 2023 across all data sources and regions.

#### Seasonal cumulative admissions rate

**Fig 5:**
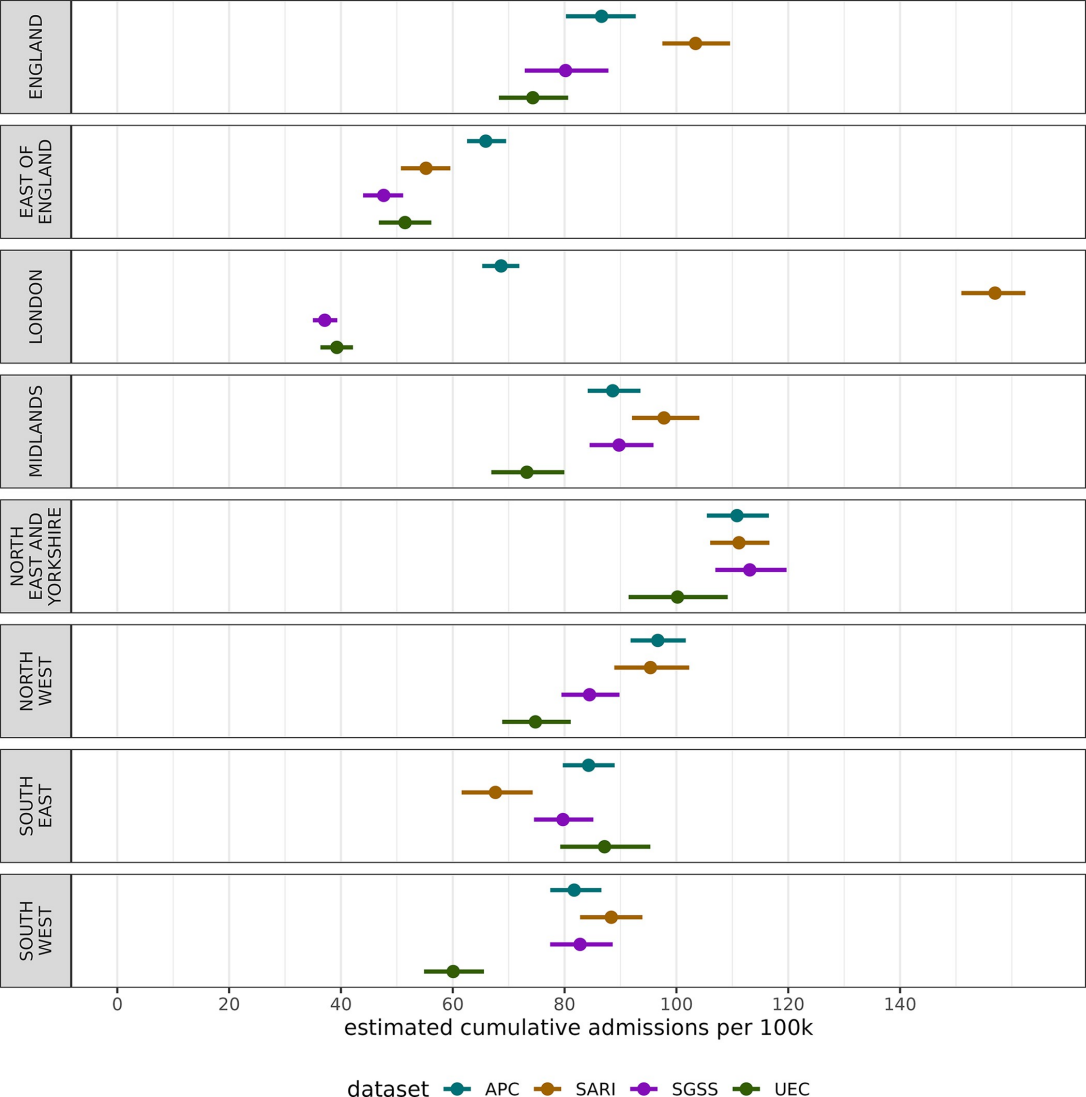
The estimated cumulative admissions per capita nationally and regionally across each data source over the 2022–2023 winter season in England. The central point represents the median estimate, and the lines the 95% confidence interval.

The burden on the healthcare system can be characterised over the whole season as the cumulative seasonal admission rates, explored in Fig 5. There is high variation in cumulative admission rate across regions and datasets. The clearest difference is the low total admissions in UEC and SGSS within London, and substantially higher rate in SARI. This London difference drives much of the national variation in cumulative rates.

#### Speed of doubling time

**Fig 6:**
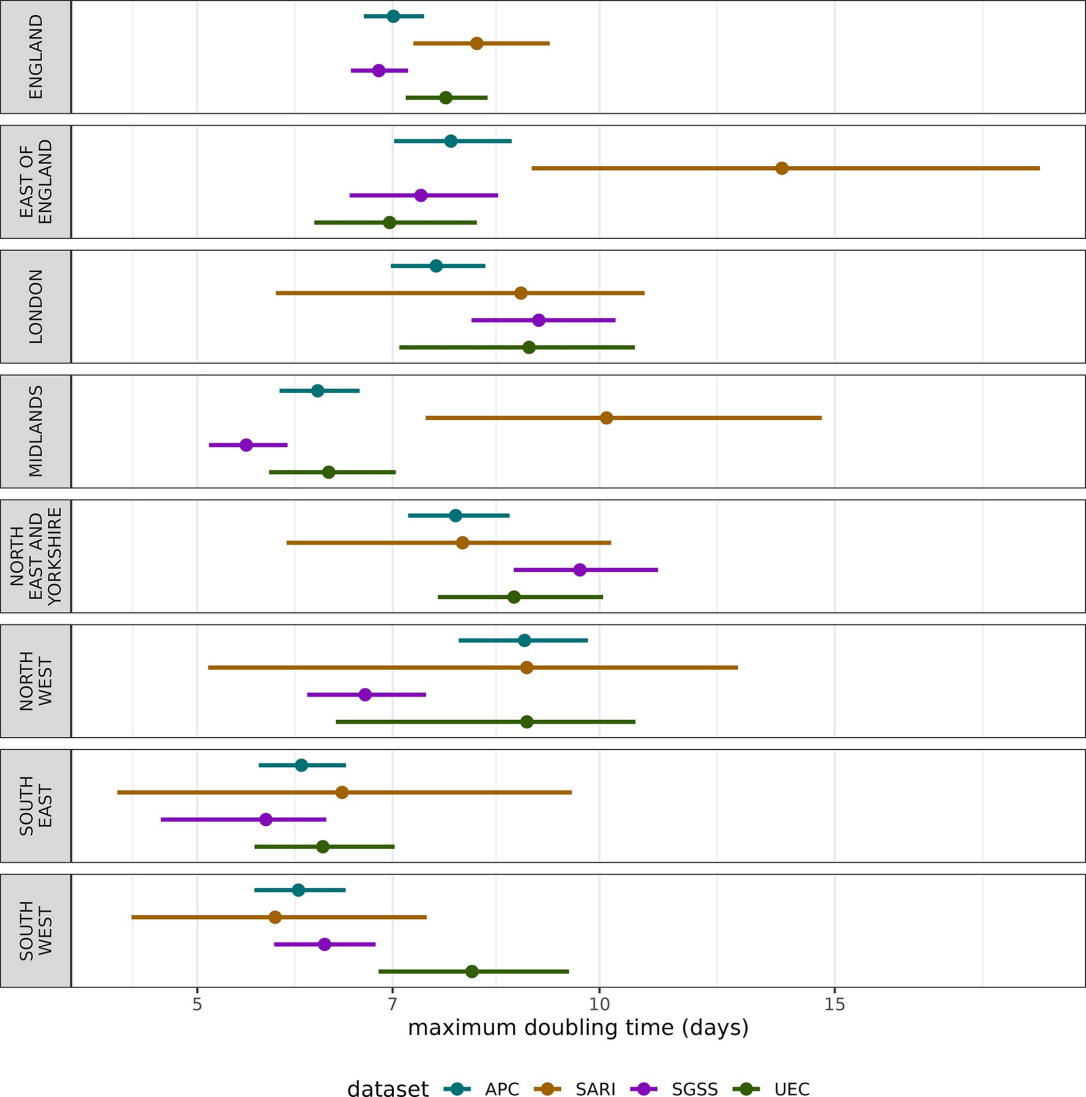
The maximum estimated doubling time (days until admissions double given the growth rate) across each data source over the 2022–2023 winter season in England. The central point represents the median estimate, and the lines the 95% confidence interval. A lower doubling time corresponds to a faster growing epidemic.

How quickly an epidemic is growing is an important metric for understanding the transmission dynamics of the virus–to explore the winter 2022–2023 influenza wave’s speed we extracted the maximum doubling time of the wave presented in Fig 6. Nationally, the epidemic wave doubled quickly, with all datasets giving a maximum doubling time of between six and nine days. Regionally, there is high variation, with some datasets giving even shorter doubling times and some substantially longer, such as SGSS and UEC in London, and SARI in East of England.

## Discussion

This research shows that while there is variation in influenza admission rates across England, this variation can be an artifact of the data or surveillance system used, rather than the epidemiology of the virus. In this work we have explored the timeliness, completeness, accuracy, and coverage of four influenza admission data sources. Regional variation in estimates influenced by surveillance sources can cause different conclusions to be drawn, despite measuring the same epidemic wave.

Across the surveillance sources, we found the highest regional variation in London and the East of England. For example, in London the UEC and SGSS data sets are at very low levels (Figs 3B, 4, 5), the sentinel SARI data indicates a large epidemic wave, whereas APC (which is retrospective and in general more complete) indicates an epidemic of a smaller magnitude. There is substantial variation in coverage for each data source across regions, particularly SGSS & SARI, as well as regional variation over time for UEC (Fig 1). The completeness relative to APC varies in London, with the sentinel approach both having under (as expected) and over completeness compared to SGSS and UEC. The findings, particularly relating to the accuracy of modelled admission rates, suggests that there are challenges in interpreting data from different regions, which naturally impact the national estimate.

At a national level there is strong agreement in trends for some metrics, such as the maximum admission rate (Fig 4) and maximum doubling time (Fig 6). However, this correspondence breaks down for the cumulative admissions rate (Fig 5), which may be more sensitive to surveillance over the whole season. Overestimates of rates may be due to samples containing Trusts that report frequently, whereas lower estimates may be due to either lower ascertainment within the reporting hospitals, or issues with the reporting itself. In effect, some areas with systemic overestimates, and others with underestimates are being averaged to produce a better national estimate–this effect cannot be relied for robust surveillance. This has important implications for monitoring the epidemic in real-time as APC is not available–the choice in dataset may lead to different conclusions in a given region. It is crucial to note, except for APC, none of the datasets have near-complete coverage of secondary care providers in England, though this is not expected of SARI which employs a sampling approach.

There are clear areas for improvement across surveillance systems. Sentinel surveillance (such as SARI) should ensure it is covering a sufficient population to be able to draw conclusions in all locations and across metrics. Data quality checks should be conducted early on rapid situational reports (such as UEC) in hospitals, with clearly erroneous data challenged, increasing the confidence in trends shown and populations represented. Administrative data sets (such as SGSS) hold promise for surveillance but require all data fields are provided consistently. Without these sampling design / data quality issues being addressed insight is lost due to biased or under sampled collection. Triangulation of different surveillance sources is critical to understanding trends [2, 37, 38]–though this is often across types of incidence [39] (such as mortality, hospitalisations, primary care) and rarely within the same incidence metric, in our case admission rates. This work highlights that increased sampling in low coverage areas and systems would improve certainty in metrics analysed, aligning with wider findings globally [40], with research highlighting the challenges of optimal population coverage for surveillance [41].

Being able to link these data sources together, to associate a positive test with a patient’s admission records would strengthen our understanding of ascertainment and the relationship between the collections. Where participation is high and data consistency is strong there is correspondence between the datasets increasing our confidence in the estimates derived. Notwithstanding London and the East of England regions, the SARI admission rates were created using a fraction of the sample size relative to other datasets. However, the SARI data provided a robust national estimate and, when correcting for day-of-week effects, a comparable regional estimate in many metrics. The higher cumulative season admission rates derived from SARI appear to be a result of higher non-peak-time values than other datasets, either representing better engagement/ascertainment in the participating Trusts, or a bias toward higher count returns.

For many regions, the results correspond, which is perhaps surprising given the different definitions for influenza admission, outlined in Table 1. While these definitions are different, their similar results imply that influenza attributable admissions were well ascertained in the 2022 season. As some influenza definitions strictly require test positivity, the inclusion of symptoms, and some clinical coding, this implies the testing for influenza is highly dependent on a symptom presentation, and clinical coding dependent on testing, rather than clinical decision making alone. We would expect higher dependence on testing used for diagnosis given the confounding effect of other respiratory illnesses over the study period, namely COVID-19. The high correspondence in clinical coding and other definitions is interesting as coding itself can be so variable; the reproducibility of clinical coding between individuals performing clinical coding is limited [42], indicating local practices will differ between Trusts. This issue is exacerbated when comorbidities, which alter the risk for influenza patients [43] are taken into account, which cause more discrepancy in coding [44].

Our findings in this study highlight the importance of surveillance systems in analysing trends, and that poor data quality in these systems can erode coverage, unnecessarily increasing uncertainty in epidemic trends. Triangulating data sources with the same metric, particularly at sub-national levels can highlight gaps and biases, helping those interpreting the data understand their limitations better. A potential extension of this research would be to produce a combined overall estimate of influenza admission rates and the relevant metrics shown here, either in real-time or post-season. This would reduce the impact of small samples in some surveillance sources and biases, providing a more robust estimate particularly in at regional levels. A variety of data and model combination approaches could be considered, though care would need to be taken regarding the different measurement schemes across data and multiple measurement of some locations across systems.

### Limitations

Comparison of data sources with different definitions of admission necessitates assumptions about their relationships, such as inpatients testing positive being an admission, which is not strictly the case. Furthermore, to compare the datasets models were fit to the data to perform inference on the different quantities of interest and adjust for different reporting frequencies. This modelling step adds a source of bias into the results as the inferences are sensitive to how models are fit.

Ideally, this analysis would be conducted across several winter waves to understand changes in datasets over time, however, there was limited seasonal influenza incidence in 2020 and 2021 in England, and not all datasets existed before this time. Future research should consider a multi-year comparison of the influenza waves across the modern datasets available and relied upon in public health.

The modelling approach used in this paper utilise population size as an offset modifying the admission count, without an associated uncertainty parameter. However, should the coverage of a system change rapidly (such as the UEC, Fig 1), this may cause sudden step-changes in the estimated admission rate over time. This sudden change may increase the uncertainty of the model smoothing functions, inflating uncertainty. This indicates highly consistent hospital participation can reduce uncertainty in estimates. As the data analysed in this study are across a range of aggregate and individual level data sets, it is not possible to assess the different sociodemographic strata included in each analysis directly. This prevents direct conclusions being drawn about bias in surveillance with these unmeasured covariates.

The results presented are conducted using after-the-fact analysis to exclude specific poor reporting Trusts, which has implications for its utility in real-time. Without the inclusion criteria the results would be substantially more biased, as increased incorrect zero reporting would drag SGSS and UEC metrics downward. If reporting quality was perfect, with admissions reported representing true admissions across datasets we would expect that the difference in incidence would be an expression only of the difference in hospital admission definition and the Trusts included in reporting, however, this quality issue prevents us from inferring the differences in definitions.

Crucially, this work only looks at one very specific part of influenza surveillance. We have not explored the utility of different data sources from a public health perspective or an overall healthcare system view, but rather specifically on a comparable metric–the admission rate per capita. This analysis quantitively explored the differences in the data for influenza admissions in England, however, there is further scope to understand each system’s properties and capabilities. This would support a health economic evaluation of influenza surveillance for hospitalisations, identifying the value add of each system.

## Conclusion

In this research we have shown that influenza admission rate trends can be sensitive to data collection and the systems used. This was shown by modelling the data to make each wave of incidence comparable in the 2022–2023 season and estimating different key epidemic metrics of interest. Though there is clear agreement between the different data sources of varying sample size and collection rigor, this relationship breaks down regionally, particularly for London and East of England data reporting. The estimated peak size, timing and growth rates are similar across data sets, though the cumulative admission rate varies substantially regionally. We show that the choice of data can clearly impact the conclusions drawn from inference of the epidemic wave.

## Supporting information

S1 Text (DOCX)
