# Supplementary material for: A comparative study of influenza surveillance systems and administrative data in England during the 2022–2023 season
Source: PLOS Glob Public Health. 2024 Sep 20;4(9):e0003627. doi: 10.1371/journal.pgph.0003627 (PMC11414916; doi:10.1371/journal.pgph.0003627)
Supplement: S1 Text — (DOCX) [file pgph.0003627.s001.docx]

**Section 1**

*
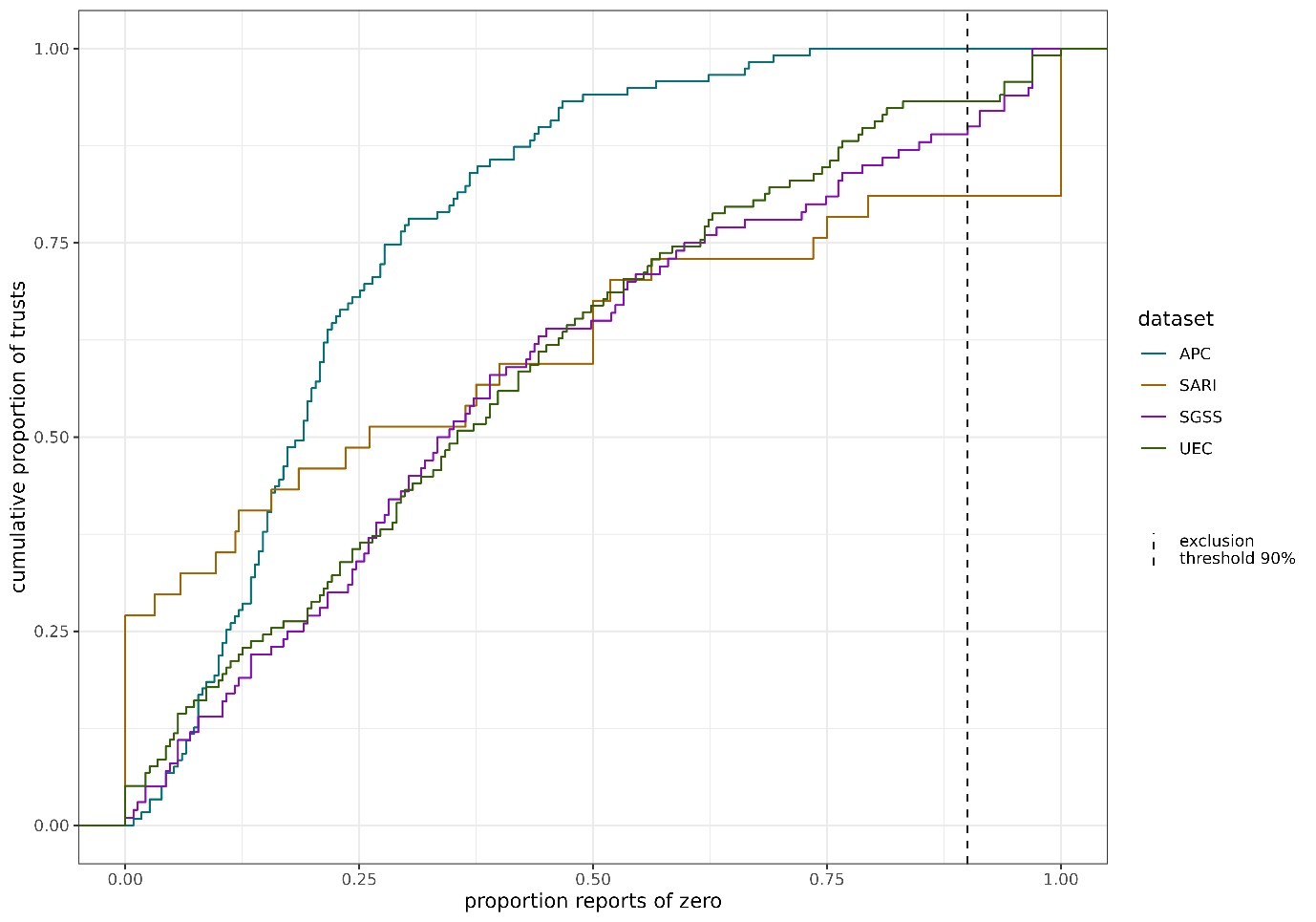
*

Fig A. The proportion of trusts which have a set proportion of zeros reported over the study period. The weekly rolling mean value of the daily data (APC, SGSS, UEC) is shown to make it comparable to the weekly SARI. The cut-off threshold in the proportion of reports is used as an exclusion criteria for misreporting. A lower threshold would exclude more trusts that may have reported correctly across data sources, while a higher threshold would have limited impact.

| **Region** | **Dataset** | **Trusts reporting (minimum – maximum)** | **Mean Population Coverage (%)** | **Peak Admissions** | **Cumulative Admissions** |
| --- | --- | --- | --- | --- | --- |
| **EAST OF ENGLAND** | **APC** | 12 - 12 | 94.6 | 117 | 3371 |
|  | **SARI** | 1 - 5 | 20.2 | 12 | 85 |
|  | **SGSS** | 7 - 7 | 50.0 | 66 | 1216 |
|  | **UEC** | 10 - 13 | 84.8 | 70 | 1829 |
| **LONDON** | **APC** | 18 - 18 | 93.3 | 223 | 6933 |
|  | **SARI** | 2 - 4 | 19.2 | 81 | 483 |
|  | **SGSS** | 12 - 12 | 63.6 | 60 | 1723 |
|  | **UEC** | 10 - 17 | 73.5 | 60 | 2032 |
| **MIDLANDS** | **APC** | 20 - 20 | 95.2 | 311 | 8787 |
|  | **SARI** | 0 - 2 | 9.0 | 29 | 151 |
|  | **SGSS** | 16 - 16 | 84.5 | 306 | 8508 |
|  | **UEC** | 15 - 18 | 81.5 | 244 | 6541 |
| **NORTH EAST AND YORKSHIRE** | **APC** | 21 - 21 | 99.1 | 292 | 10070 |
|  | **SARI** | 1 - 3 | 17.1 | 41 | 229 |
|  | **SGSS** | 13 - 13 | 69.9 | 224 | 7516 |
|  | **UEC** | 15 - 19 | 80.5 | 251 | 8148 |
| **NORTH WEST** | **APC** | 16 - 16 | 88.8 | 230 | 6648 |
|  | **SARI** | 3 - 4 | 11.7 | 23 | 106 |
|  | **SGSS** | 13 - 13 | 59.2 | 186 | 3966 |
|  | **UEC** | 11 - 13 | 71.1 | 139 | 4622 |
| **SOUTH EAST** | **APC** | 15 - 15 | 88.4 | 218 | 5724 |
|  | **SARI** | 1 - 3 | 10.1 | 12 | 60 |
|  | **SGSS** | 8 - 8 | 50.4 | 177 | 3277 |
|  | **UEC** | 12 - 14 | 84.7 | 318 | 6505 |
| **SOUTH WEST** | **APC** | 13 - 13 | 100.0 | 206 | 4510 |
|  | **SARI** | 1 - 3 | 16.6 | 37 | 122 |
|  | **SGSS** | 9 - 9 | 68.2 | 145 | 3400 |
|  | **UEC** | 10 - 12 | 84.6 | 133 | 3029 |

Table A. Unmodelled NHS regional summaries of reported data across the different data sources. Counts are from the processed data after exclusion criteria are applied. Trust counts are the lowest and highest number of participating Trusts for a given report post exclusion criteria. The mean population coverage is taken as the mean national proportion of the population over the time series. Peak admissions are taken as the maximum admissions in each report and cumulative admissions the sum of all admissions reported. These metrics are not corrected for time varying participation and population catchment sizes.


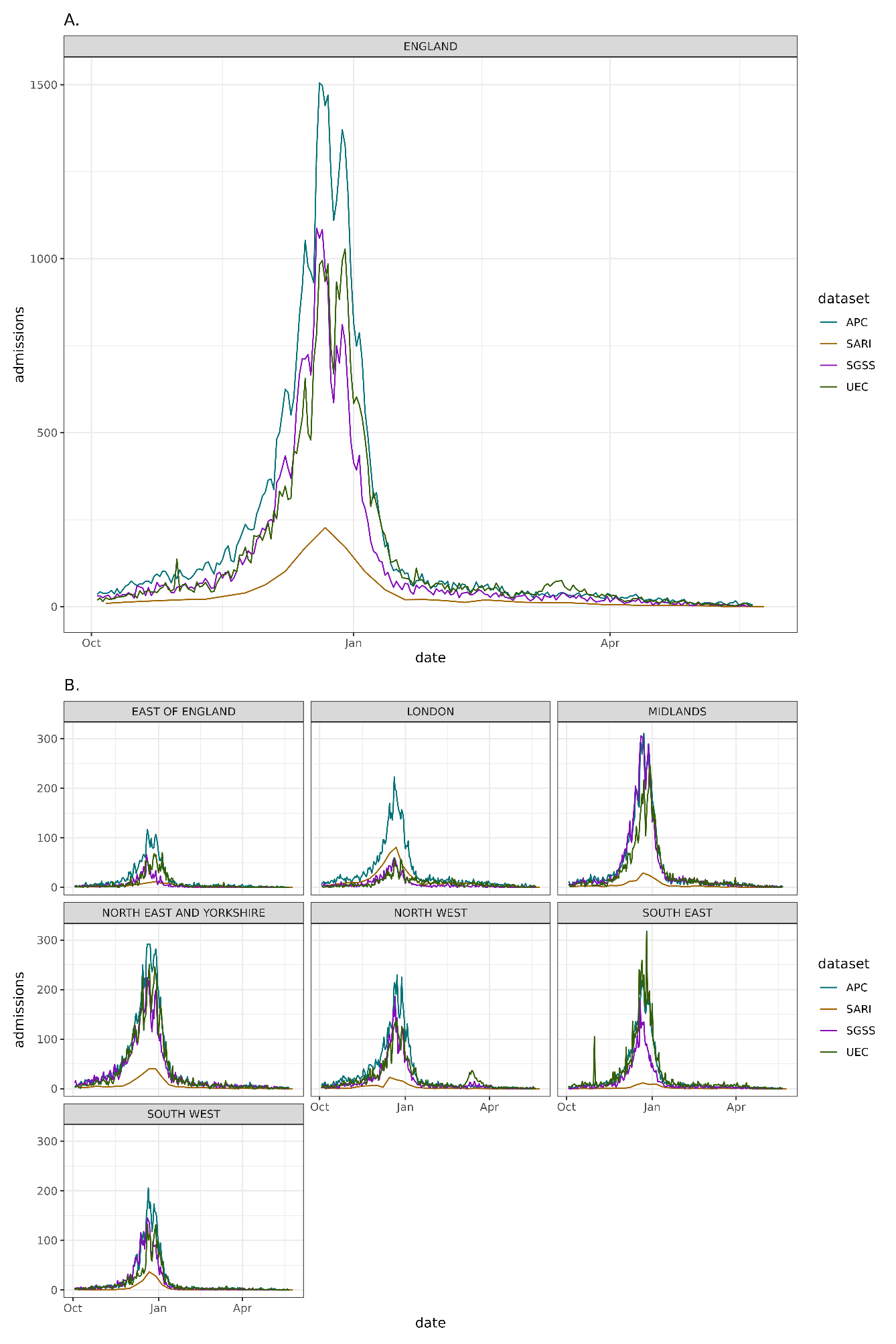


*Fig B. Influenza admission count nationally (sub-plot A) and NHS commissioning region (sub-plot B) over the winter 2022/23 season.*


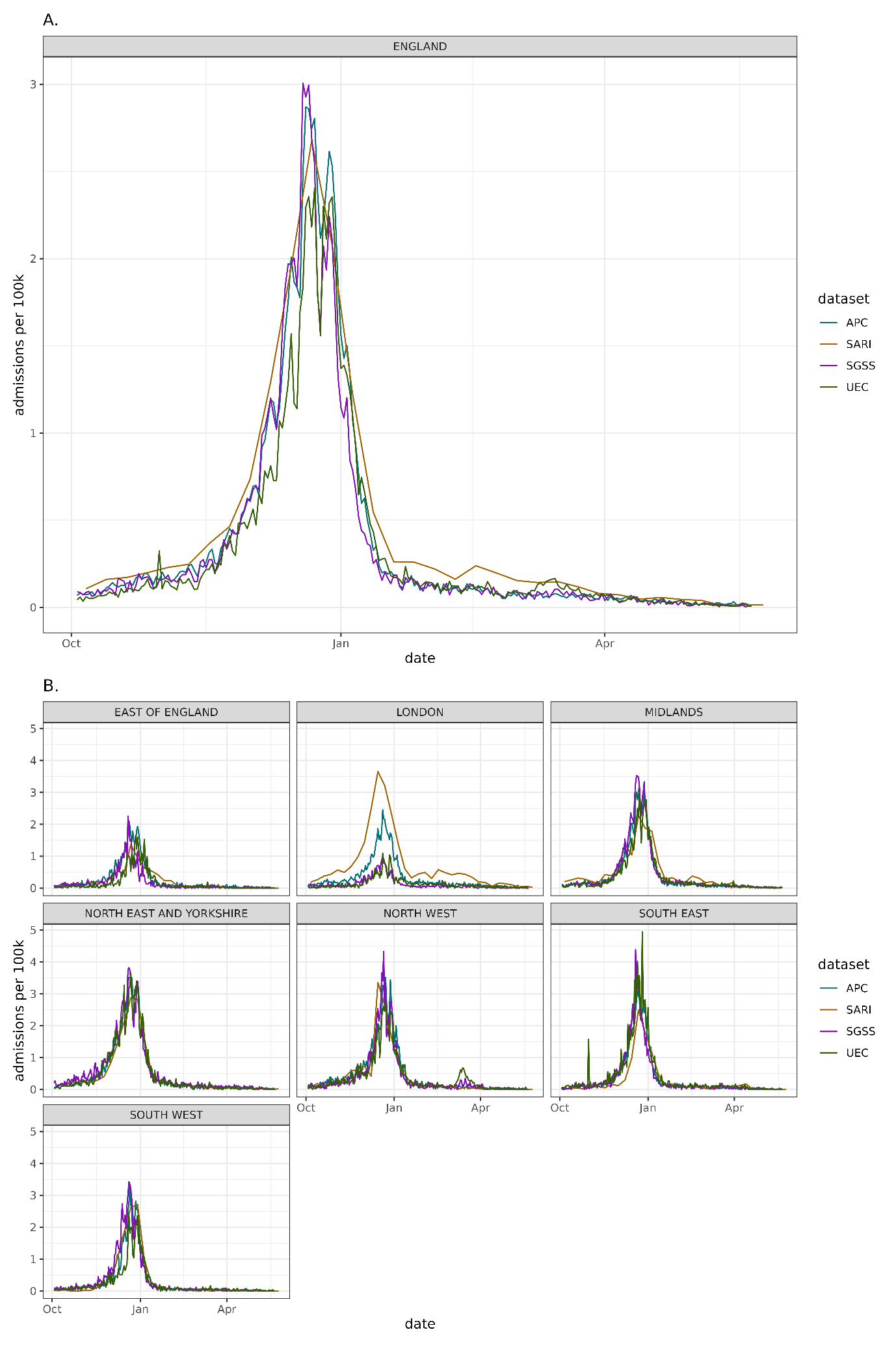
*Fig C. Influenza admission rate per 100k trust catchment population nationally (sub-plot A) and NHS commissioning region (sub-plot B) over the winter 2022/23 season.*


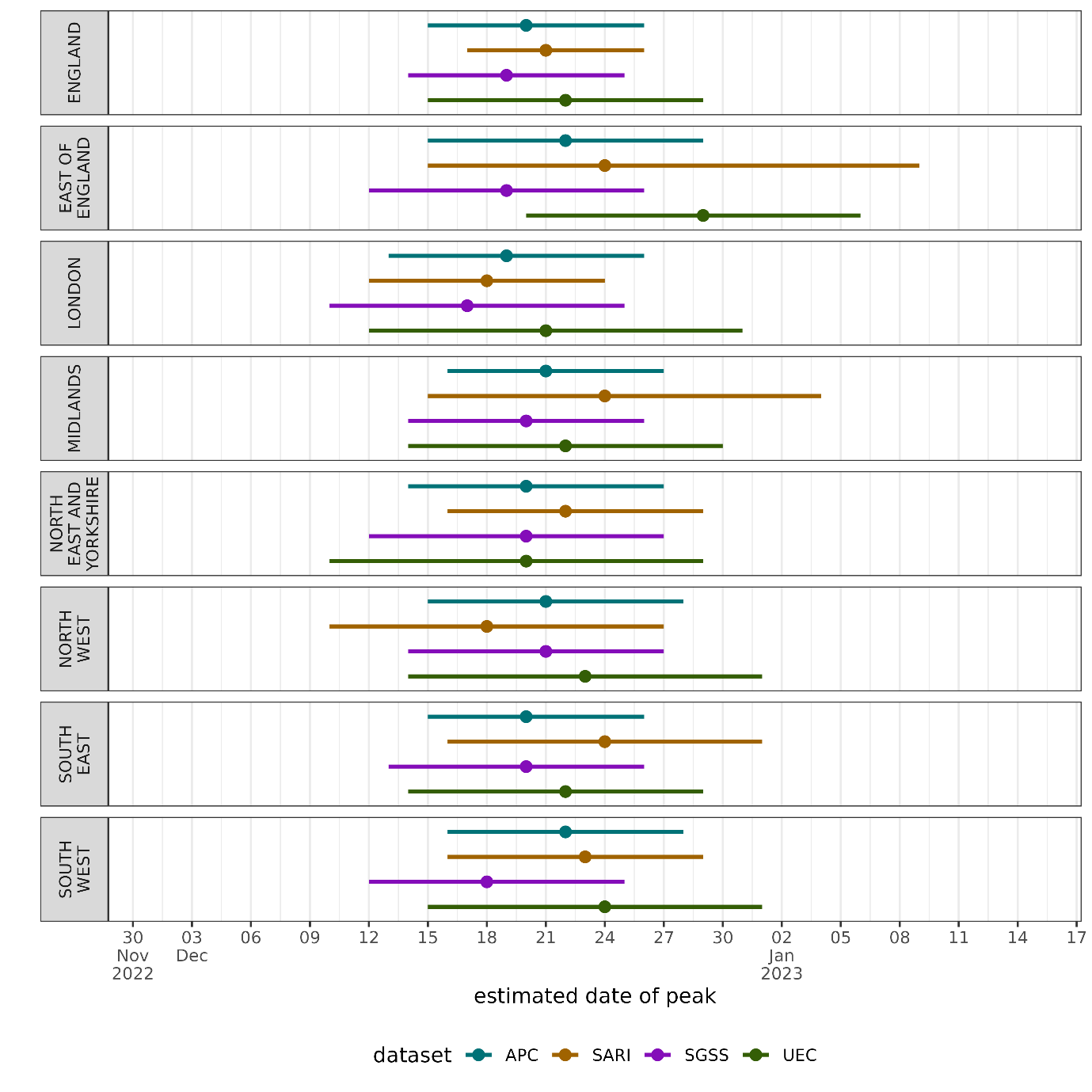


Fig D. The estimated date of the epidemic wave peaking nationally and regionally across each data source in the winter 2022/23 influenza season. The central point represents the median estimate, and the lines the 95% confidence interval.
